# Eyelid Juvenile Xanthogranuloma: A Case Report and Literature Review

**DOI:** 10.7759/cureus.5008

**Published:** 2019-06-26

**Authors:** Amirah Hassan, Norlaila Talib, Sellymiah Adzman, Adil Hussein

**Affiliations:** 1 Ophthalmology, Hospital Universiti Sains Malaysia, Kota Bharu, MYS; 2 Ophthalmology, Hospital Serdang, Selangor, MYS; 3 Pathology, Hospital Serdang, Selangor, MYS; 4 Ophthalmology, School of Medical Sciences, Universiti Sains Malaysia, Kubang Kerian, MYS

**Keywords:** eyelid swelling, juvenile xanthogranuloma, touton giant cell

## Abstract

Juvenile xanthogranuloma (JXG) is an uncommon condition affecting the eye. We herein report a rare case of eyelid swelling in paediatric age group. A three-year-old Malay boy presented with chronic painless left upper eyelid mass which did not resolve with topical steroid. Clinically, the mass was a non-tender and firm nodular swelling which located at the lateral 1/3 of the left upper lid. Total excisional biopsy of the swelling was done and histopathological findings were consistent with JXG. The systemic associations and the treatment options for ocular JXG are discussed.

## Introduction

Juvenile xanthogranuloma (JXG) is a benign histiocytosis characterized by histologic findings of lipid-laden histiocytes and giant cells [1]. It is the most common type of non-Langerhans histiocytosis that commonly affects the skin [1, 2]. The first ocular JXG that involved the iris was reported in 1948 at a meeting of the Ophthalmic Pathology Club in Washington, DC and subsequently published by Blank et al. one year later [3]. JXG of the eye has become an eye-opener in ophthalmology when 15 eyes were enucleated but 13 eyes were misdiagnosed with malignant intraocular tumor in a multicentre series of 20 cases of iris JXG [4]. We report here an interesting and rare case of eyelid JXG diagnosed in paediatric patient.

## Case presentation

A three-year-old Malay boy presented to the ophthalmology clinic with painless left upper eyelid mass for six months duration which gradually increased in size. Initially the small mass started at lateral 1/3 of the left upper eyelid. He denied any itchiness, redness, or discharge from the lesion. There was no history of similar presentation before. He was able to open his eye and the eyelid mass did not obstruct his vision. He denied any history of trauma, insect bite, or blurring of vision. The parents neither used traditional medication nor sought treatment prior to the presentation. In view of progressive growth of the mass which obstructed his lateral vision, he was brought to hospital to seek immediate treatment.

**Figure 1:**
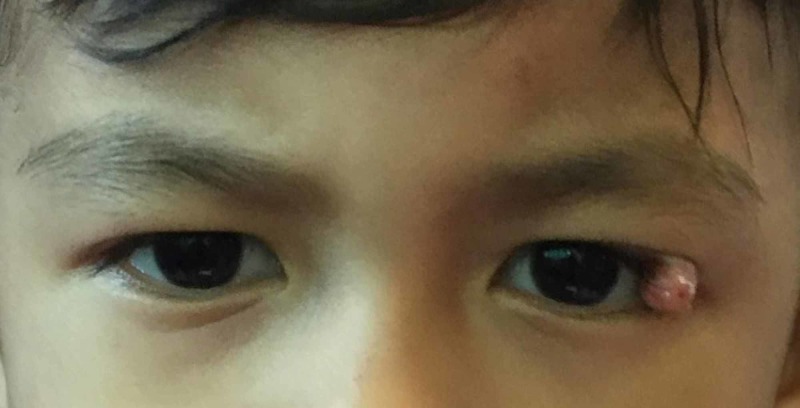
A pedunculated yellowish nodule at left upper eyelid.

On examination, the vision for both eyes were 6/6 with no relative afferent pupillary defect. There was a left upper eyelid mass at the lateral 1/3 which was 1 x 1 cm, non-erythematous, nodular, and mobile from underlying structure (Figure 1). The mass was non-tender and firm. The eyes were symmetrical, no proptosis and orthophoric in primary position. The conjunctiva was white and no mass noted. The cornea was clear and there was no hyphema. The iris was normal in colour and no iris heterochromia or iris nodule presented. Intraocular pressure was within normal range. The fundus revealed normal findings with no optic disc swelling or gliosis. He was initially treated with topical steroid. However, the mass did not resolve and he underwent excisional biopsy of the swelling of upper eyelid under general anaesthesia.

**Figure 2:**
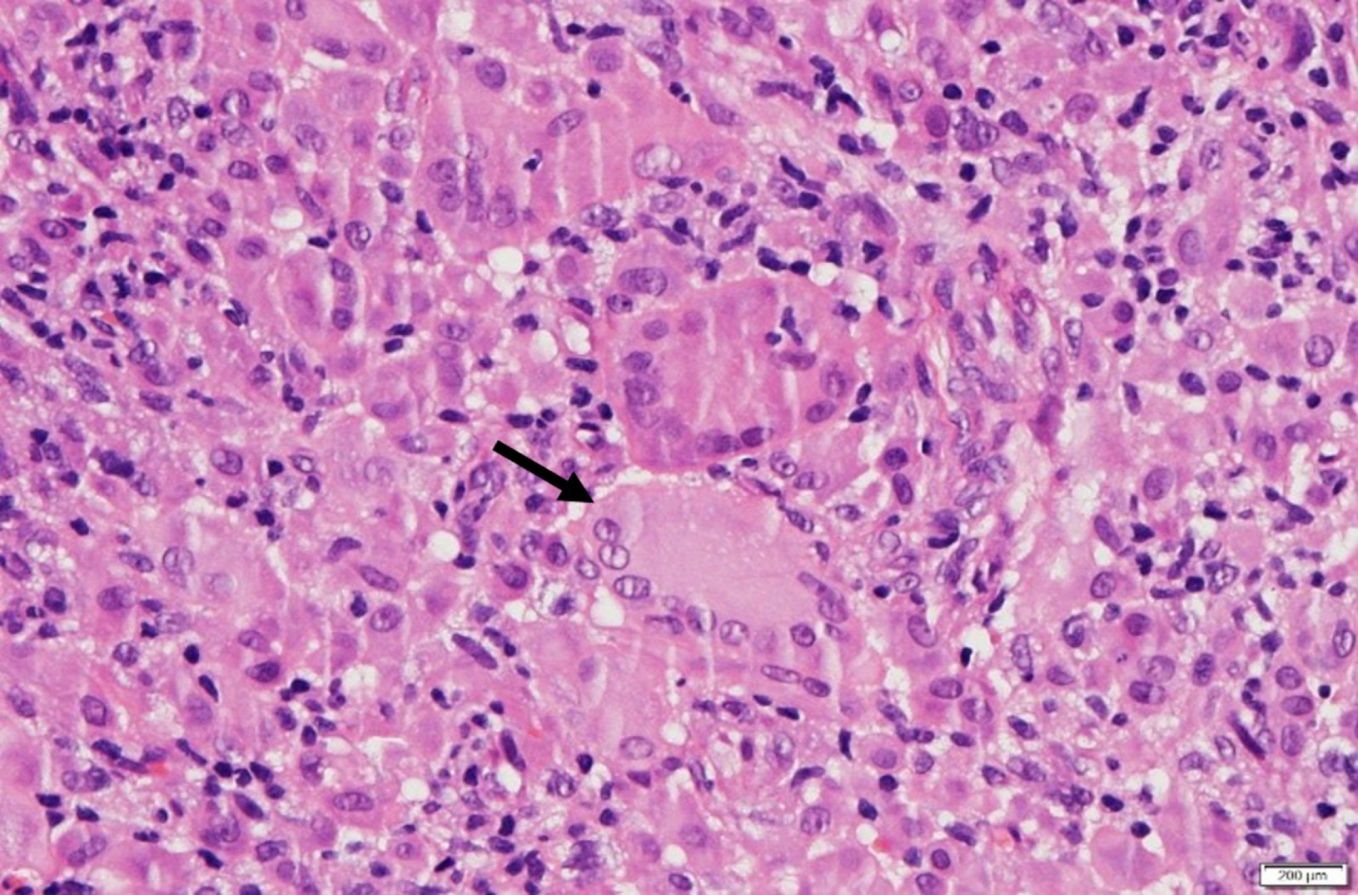
Histopathological examination of excision biopsy showing the presence of touton giant cell (arrow) with inflammatory cells.

The histopathology examination revealed a nodular lesion in the subepithelium composed of dense infiltration of lymphocytes, histiocytes, touton giant cells with some neutrophils and fibrous tissue seen amongst the inflammatory cells (Figure 2). The findings were interpreted as juvenile xanthogranuloma (JXG). The patient was referred to paediatric unit to exclude systemic involvement of juvenile xanthogranuloma and it was confirmed that there was no systemic involvement present. At three months follow-up the swelling did not recur after the surgery and the surgical site healed with a faint scar.

## Discussion

Juvenile xanthogranuloma is a rare manifestation of ocular disease. The incidence of eye involvement in patients with cutaneous JXG was estimated to be 0.3% to 0.4%. In contrast, at least 41% of patients with ocular involvement had cutaneous lesions and they are always multiple in number [5]. Ocular involvement occurs more often during the first two years of life, but adult onset is also observed [5, 6].

Iris is the most common ocular site for JXG. Iris JXG is usually asymptomatic but it can present with hyphema, glaucoma, erythema with signs of uveitis, or congenital/acquired heterochromia iridis [1]. The eyelid is the second common site for ocular JXG while the posterior segment JXG is very rare [7]. Orbital involvement is also unusual and appears to occur mainly during the perinatal period [1, 7, 8].

Diagnosis of JXG is mainly by clinical histopathology. The tissue or sample is obtained by excision biopsy, paracentesis, iridectomy, or intravitreal sampling. Most of ocular JXG is treated either conservatively or with medical treatment rather than surgical intervention. In non-sight threatening condition, it can be treated with high-dose topical steroid, periocular steroid and systemic corticosteroid [2]. There has been reports of successfully treating limbal JXG using topical corticosteroids in a four-month-old male patient, and also using intralesional corticosteroid for congenital eyelid JXG in an 18-day-old infant [9, 10].

In our patient, he was treated with topical steroid for two weeks. However, the swelling did not improve and thus excisional biopsy was planned as a diagnostic and therapeutic management for this patient. He was referred to paediatric team to rule out systemic involvement. It is important to realize the relationship of JXG with neurofibromatosis type I (NF1) and juvenile myelomonocytic leukemia (JMML). Zvulunov et al. studied this association and concluded that children with NF1 and JXG have a 20- to 30-fold higher risk for JMML than patients with NF1 without JXG [11]. On the other hand, children with newly diagnosed JXG, multiple skin lesions, and onset at two years or younger were found to be at greatest risk and should therefore be targeted for surveillance [5].

Table 1 shows published cases of ocular and orbital JXG. Glaucoma and hyphema are likely to develop when JXG involved the iris. Newell summarized that spontaneous hyphema can occur in ocular JXG which is self-limiting [12]. Early recognition is possible to prevent loss of the eye during acute stage of the disease. Based on the case reports, most of JXG that involved deep structures of the eye such as iris, retina or choroid are usually associated with systemic manifestation. Gharib et al. and Meyer et al. performed cutaneous biopsy as a guidance to diagnosed spontaneous hyphema [13, 14]. They successfully preserved the eye by treating the disease conservatively.

**Table 1:** Summary of published case reports of ocular JXG. JXG: Juvenile xanthogranuloma

Authors	Year	Age	Sex	Ocular presentation	Systemic manifestation	Treatment	Outcome
Blank et al. [3]	1948	Four months old	Male	Glaucoma secondary iris JXG	Numerous JXG on head and trunk	Enucleation	Healthy after 11 months with spontaneous resolution
Gharib et al. [13]	1956	Four months old	Female	Left eye hyphema secondary iris JXG	Patchy edematous lesion on neck, face, and arms	Conservative biopsy taken from skin lesion	Resolution of hyphema skin lesion after seven months
Newell [12]	1957	10 months old	Female	Left eye glaucoma secondary iris JXG	Multiple yellowish plaque on back, chest, and head	Biopsy and large peripheral iridectomy	Not reported
		2 ½ months old	Female	Left eye hyphema secondary to iris, ciliary body, and trabeculum JXG	No systemic manifestation reported	Enucleation	Not reported
Gass JDM [15]	1964	nine months old	Female	JXG of right iris and ciliary body with hyphema, chronic secondary glaucoma, vitreous hemorrhage, and edema and detachment of the macula	Not reported	Enucleation	Not reported
		22 months old	Female	Right eye glaucoma secondary to iris JXG	Not reported	Iridectomy and topical steroid	No recurrence after 2 ½ years
Wertz et al. [16]	1982	20 months old	Female	Right neovascular glaucoma secondary to optic nerve, disc, retina and choroid JXG	No systemic involvement	Enucleation	No recurrence of systemic involvement
DeBarge et al. [7]	1994	12 years old	Male	Right eye uveitis secondary to chorioretinal, iris, and ciliary body JXG	Not reported	Iris biopsy and paracentesis intraocular steroid	Residual iris/ciliary body involvement resolved
Viola et al. [17]	2004	11 months old	Female	Left JXG of optic disc and retina	Facial and eyelid JXG	Presumed from skin lesion systemic steroid	Retinal detachment and vitreous haemorrhage 20 months after treatment
Hayashi et al. [18]	2004	31 months old	Female	Left upper eyelid JXG	No systemic involvement	Excision biopsy	No recurrence
Kuruvilla et al. [10]	2009	18 months old	Not documented	Right upper eyelid JXG	No systemic involvement	Incisional biopsy and intralesional steroids	Resolution of the remaining lesion after four weeks
Johnson et al. [8]	2010	Six weeks old	Male	Right JXG of orbit	JXG of sinuses, brain, and subtemporal fossa	Conservative biopsy from bone marrow biopsy	Resolution after 18 months
Carol et al. [19]	2015	Three months old	Female	Left upper eyelid JXG	No systemic involvement	Incisional biopsy and oral steroids	No recurrence after five months
Meyer et al. [14]	2018	Four months old	Female	Left eye hyphema and glaucoma secondary to iris JXG	Yellowish brown papules on abdomen	Topical steroids	Left eye amblyopia

## Conclusions

Simple eyelid swelling due to JXG may associate with systemic involvement. The crucial parts in managing eyelid swelling are histopathological finding and identification of ocular and systemic association to prevent inevitable complications. In uncomplicated cases, conservative globe-sparing treatment has shown as the best option for tumor resolution with low incidence of recurrence.
